# Regulatory role of CD8^+ ^T lymphocytes in bone marrow eosinophilopoiesis

**DOI:** 10.1186/1465-9921-7-83

**Published:** 2006-06-01

**Authors:** Madeleine Rådinger, Svetlana Sergejeva, Anna-Karin Johansson, Carina Malmhäll, Apostolos Bossios, Margareta Sjöstrand, James J Lee, Jan Lötvall

**Affiliations:** 1 Lung Pharmacology Group, Department of Internal Medicine/Respiratory Medicine and Allergology, Göteborg University, Göteborg, Sweden; 2 The Unit for Lung Investigations, Faculty of Science, Department of Gene Technology, Tallinn University of Technology, Estonia; 3 Divison of Pulmonary Medicine, Mayo Clinic, Scottsdale, AZ 85259, USA

## Abstract

**Background:**

There is a growing body of evidence to suggest that CD8^+ ^T lymphocytes contribute to local allergen-induced eosinophilic inflammation. Since bone marrow (BM) responses are intricately involved in the induction of airway eosinophilia, we hypothesized that CD8^+ ^T lymphocytes, as well as CD4^+ ^T lymphocytes, may be involved in this process.

**Methods:**

Several approaches were utilized. Firstly, mice overexpressing interleukin-5 (IL-5) in CD3^+ ^T lymphocytes (NJ.1638; CD3^IL-5+ ^mice) were bred with gene knockout mice lacking either CD4^+ ^T lymphocytes (CD4^-/-^) or CD8^+ ^T lymphocytes (CD8^-/-^) to produce CD3^IL-5+ ^knockout mice deficient in CD4^+ ^T lymphocytes (CD3^IL-5+^/CD4^-/-^) and CD8^+ ^T lymphocytes (CD3^IL-5+^/CD8^-/-^), respectively. Secondly, CD3^+^, CD4^+ ^and CD8^+ ^T lymphocytes from naïve CD3^IL-5+ ^and C57BL/6 mice were adoptively transferred to immunodeficient SCID-bg mice to determine their effect on BM eosinophilia. Thirdly, CD3^IL-5+^, CD3^IL-5+^/CD8^-/- ^and CD3^IL-5+^/CD4^-/- ^mice were sensitized and allergen challenged. Bone marrow and blood samples were collected in all experiments.

**Results:**

The number of BM eosinophils was significantly reduced in CD3^IL-5+^/CD8^-/- ^mice compared to CD3^IL-5+ ^mice and CD3^IL-5+^/CD4^-/- ^mice. Serum IL-5 was significantly higher in CD3^IL-5+^/CD4^-/- ^mice compared to CD3^IL-5+ ^mice but there was no difference in serum IL-5 between CD3^IL-5+^/CD4^-/- ^and CD3^IL-5+^/CD8^-/- ^mice. Adoptive transfer of CD8^+^, but not CD4^+ ^T lymphocytes from naïve CD3^IL-5+ ^and C57BL/6 mice restored BM eosinophilia in immunodeficient SCID-bg mice. Additionally, allergen challenged CD3^IL-5+^/CD8^-/- ^mice developed lower numbers of BM eosinophils compared to CD3^IL-5+ ^mice and CD3^IL-5+^/CD4^-/- ^mice.

**Conclusion:**

This study shows that CD8^+ ^T lymphocytes are intricately involved in the regulation of BM eosinophilopoiesis, both in non-sensitized as well as sensitized and allergen challenged mice.

## Background

One important pathologic feature of allergic airway inflammation is associated with T lymphocyte activation and increase in eosinophil numbers in the airways [1-3]. Accumulation of eosinophils is considered to be the result of increased production and traffic of cells from the bone marrow (BM) into the airways via the circulation [4,5]. A substantial body of evidence suggests that BM eosinophilopoiesis is enhanced in allergic patients as well as in animal models of allergen-induced inflammation [6-13].

The allergen-induced increase in eosinophil numbers is closely linked to a Th_2 _driven immune response based on the specific expression of cytokines exclusively secreted from CD4^+ ^T lymphocytes [2,3]. In particular, the expression of interleukin-5 (IL-5) by T lymphocytes has been shown to be an essential signal necessary for the induction of eosinophilia in the airway [4,5,14-17].

Whereas the pivotal role of CD4^+ ^T helper (Th) cells in the development of allergic diseases has been demonstrated in several models, the exact role of CD8^+ ^T lymphocytes remains unclear. Generally, the CD8^+ ^T lymphocytes are considered to produce Th_1 _cytokines, which is not always the case, since under certain circumstances CD8^+ ^T lymphocytes also can produce Th_2 _cytokines. For example, CD8^+ ^T lymphocytes have been shown to produce IL-4, IL-5 and IL-13 following allergen stimulation [17-20].

An increasing amount of data suggests that CD8^+ ^T lymphocytes contribute to allergen-induced airway inflammation. Depletion of CD8^+ ^T lymphocytes prior to allergen challenge has been shown to decrease Th_2 _cytokines, reduce eosinophil recruitment into the airway and reduce airway hyperresponsiveness [19-22]. Although CD8^+ ^T lymphocytes appear to be involved in the regulation of local airway inflammation, less is known about their putative role in regulating distant pro-inflammatory responses, such as the enhanced eosinophilopoiesis seen after allergen exposure. We hypothesized that IL-5 producing CD8^+ ^T lymphocytes may regulate BM responses following airway allergen exposure. To test this, we utilized an IL-5 transgenic mouse overexpressing IL-5 in CD3^+ ^T lymphocytes (NJ.1638; CD3^IL-5+^) that was bred with gene knockout mice lacking either CD4^+ ^cells (CD4^-/-^) or CD8^+ ^cells (CD8^-/-^) in order to produce IL-5 transgenic-gene knockout mice deficient in CD4^+ ^and CD8^+ ^T lymphocytes, respectively. Bone marrow and blood samples were taken from offspring as well as from CD3^IL-5+ ^mice. Additionally, CD3^+^, CD4^+ ^or CD8^+ ^T lymphocytes from naïve CD3^IL-5+ ^and wild type C57BL/6 mice were adoptively transferred to immunodeficient SCID-bg mice, in order to determine their role in regulating BM eosinophilia.

## Methods

### Mice

IL-5 transgenic mice (NJ. 1638 (CD3^IL-5+^)) overexpressing IL-5 specifically in CD3^+ ^T lymphocytes were obtained from Dr James J Lee (Mayo Clinic, Scottsdale, AZ, USA) and maintained in a heterozygous fashion by back-crossing to C57BL/6 mice. CD3^IL-5+ ^mice were bred with gene knockout mice lacking either CD4^+ ^T lymphocytes (C57BL/6J CD4^tm1Knw^) or CD8^+ ^T lymphocytes (C57BL/6 CD8a^tm1Mak^) (Jackson Laboratories, Bar Harbor, ME) to produce CD3^IL-5+ ^knockout mice deficient in CD4^+ ^and CD8^+ ^T lymphocytes, respectively. Genotypes of mice produced by this crosses were assessed by the presence of CD3^IL-5+ ^and loss of T lymphocyte subtypes (PCR of tail DNA). Briefly, DNA was isolated from tail biopsies by using the DNeasy Tissue kit according to the manufacturer's instructions (Qiagen, Crawley, UK). The PCR reactions of DNA from C57BL/6 CD4^tm1Knw ^and C57BL/6 CD8a^tm1Mak ^were prepared using the HotStartTaq Master Mix Kit (Qiagen, Crawley, UK) according to the protocol received from The Jackson Laboratory (Jackson Laboratories, Bar Harbor, ME). The PCR reactions of CD3^IL-5+ ^were assessed as previously described with some modifications [23].

Wild type C57BL/6 mice and C.B-17/Gbms Tac-SCID-bg mice were purchased from Mollegaard-Bommice A/S (Ry, Denmark). SCID-bg mice are immunodeficient mice that lack functional B and T-lymphocytes. All mice were provided with food and water *ad libitum *and housed in specific pathogen free animal facilities. The study was approved by the Ethics Committee for animal studies in Göteborg, Sweden.

### Sample collection and processing

The animals were euthanized with a mixture of xylazin (130 mg/kg, Rompun^®^, Bayer) and ketamine (670 mg/kg, Ketalar^®^, Parke-Davis). First, blood was obtained by puncture of the heart right ventricle. Second, bronchoalveolar lavage (BAL) was performed by instilling 0.5 ml of phosphate buffered saline (PBS) through the tracheal cannula, followed by gentle aspiration and repeated with 0.5 ml PBS. Finally, bone marrow was harvested by excising one femur, which was cut at the epiphyses and flushed with 2 ml of PBS.

#### Blood

Two hundred microliters of blood was mixed with 800 μl of 2 mM EDTA (Sigma-Aldrich) in PBS, and red blood cells (RBC) were lysed in 0.1% potassium bicarbonate and 0.83% ammonium chloride for 15 min at RT. White blood cells (WBC) were resuspended in PBS containing 0.03% Bovine serum albumin (BSA, Sigma-Aldrich). For measurement of cytokines in serum the remaining volume of blood was centrifuged at 800 g for 15 min at 4°C.

#### Bone Marrow and Bronchoalveolar lavage fluid (BALF)

BM and BALF samples were centrifuged at 300 g for 10 min at 4°C. The cells were resuspended with 0.03% BSA in PBS. The total cell numbers in blood, BM and BALF were determined using standard hematological procedures. Cytospins of blood, bone marrow and BALF samples were prepared and stained according to the May-Grünwald-Giemsa method for differential cell counts. Cell differentiation was determined by counting 300–500 cells using a light microscope (Zeiss Axioplan 2, Carl Zeiss, Jena, Germany). The cells were identified using standard morphological criteria.

### Sensitization and allergen exposure and in vivo labeling of newly produced eosinophils

Mice, 8–12 weeks old were sensitized on two occasions, five days apart by intraperitoneal (i.p) injections of 0.5 ml alum-precipitated antigen containing 8 μg Ovalbumin (OVA) (Sigma-Aldrich, St Louis, MO, USA) bound to 4 mg of Al(OH)_3 _(Sigma-Aldrich) in PBS. Eight days after the second sensitization, the mice were rapidly and briefly anaesthetized with Isoflourane (Schering-Plough, UK), and received intranasal (i.n.) administration of 10 μg OVA in 25 μl PBS during five consecutive days. Twenty-four hours after the last OVA exposure the mice were sacrificed and cells from blood, BM and BALF were collected as described above. Additionally, the animals were given 5-Bromo-2'-deoxyuridine (BrdU) (Roche, Diagnostics Scandinavia AB, Bromma, Sweden) to label newly produced eosinophils. The BrdU was given at a dose of 1 mg in 250 μl PBS by i.p. injection twice, 8 hours apart on day 1 and on day 3 during OVA exposure.

### Double immunostaining for nuclear BrdU and Major Basic Protein (MBP)

On day 1, cytospin preparations were fixed in 2% formaldehyde for 10 min and incubated with 10% rabbit serum (DAKO Corporation, Glostrup, Denmark) to avoid unspecific binding. BM and BALF slides were incubated with a monoclonal rat anti-mouse MBP antibody (kind gift from Dr James J Lee, Mayo Clinic, Scottsdale, AZ) for 1 hour followed by a 45 min incubation with alkaline phosphatase-conjugated rabbit F(ab')_2 _anti-rat IgG secondary antibody (DAKO). Bound antibodies were visualized with Liquid Permanent Red substrate kit (DakoCytomation Inc, Carpenteria, CA, USA). Samples were fixed for a second time over night in 4% paraformaldehyde. On day 2, samples were treated with 0.1% trypsin (Sigma) at 37°C for 15 min followed by 4 M HCl for 15 min and Holmes Borate buffer (pH 8.5) for 10 min. Endogenous peroxidase was blocked with glucose oxidase solution (PBS supplemented with 0,0064% sodium azide, 0,18% glucose, 0,1% saponin and 1.55 units of glucose oxidase/ml PBS) preheated to 37°C for 30 min. BrdU labeled cells were detected using a FITC conjugated rat anti-mouse BrdU monoclonal antibody (clone BU1/75, Harlan-Sera Lab, Loughborough, UK), followed by a peroxidase conjugated rabbit anti-FITC secondary antibody (DAKO) and visualized with 3,3'-diaminobenzidine (DAB) substrate Chromogene System (DAKO). Mayer's Hematoxylin (Sigma) was used for counterstaining. Cells were determined by counting 400 cells using a light microscope (Zeiss Axioplan 2, Carl Zeiss, Jena, Germany).

### Preparation of lymphocytes

Spleens were collected from naïve CD3^IL-5+ ^or C57BL/6 mice, washed in 2% penicillin/streptomycin in PBS (Gibco BRL, Paisley, Scotland) and homogenized in 1% penicillin/streptomycin in PBS by homogenizer (POLYTRON^R ^PT 1200, Kinematica AG, Switzerland). Undigested tissue was removed by filtration through a 70-μm-nylon mesh (BD Biosciences). RBC were lysed using 0.1% potassium bicarbonate and 0.83% ammonium chloride solution for 15 minutes at 4°C and WBC were washed and re-suspended in 0.5% BSA/PBS. CD3^+^, CD4^+ ^or CD8^+ ^lymphocytes were separated by labeling spleen cells with a biotinylated hamster-anti mouse CD3ε monoclonal antibody (mAb, clone 145-2C11), a biotinylated rat-anti mouse L3T4 mAb (clone H129.19) or a biotinylated rat-anti mouse Ly-2 mAb (clone 53-6.7, all obtained from BD Biosciences). After washing, streptavidin magnetic microbeads (MACS, Miltenyi Biotec GmbH, Germany) were added according to the manufacturer's instructions. Lymphocyte subsets were enriched over a magnetic field. The purity of the enriched lymphocyte subset fractions was analyzed by FACS.

### Adoptive transfer experiments

#### Preliminary time-course experiments

CD3^+ ^lymphocytes from CD3^IL-5+ ^mice (10^7 ^cells in 0.35 ml 0.9% NaCl) or 0.9% NaCl alone was injected i.v to SCID-bg mice. Recipients were sacrificed on day 3, 10, 14, 21, 30 or 39 after cell transfer. Eosinophil numbers in BM and blood are shown in Table 1. In the final adoptive transfer experiments CD4^+^, CD8^+ ^or CD3^+ ^lymphocytes (10^7^) from CD3^IL-5+ ^or C57BL/6 mice in 0.35 ml of 0.9% NaCl or 0.9% NaCl alone was injected i.v to SCID-bg mice. All samples were obtained on day 39 after the transfer, which was based upon the most pronounced changes in BM and blood eosinophil numbers in the time-course experiment.

**Table 1:** Eosinophil numbers in SCID bg mice.

**Recipients of**	**Bone Marrow (% of total cells)**	**Blood (×10^4^/ml)**
**0.9% NaCl**		
7 days (n = 4)	1.125 ± 0.375	0.6 ± 0.1
21 days (n = 5)	0.75 ± 0.26	0.07 ± 0.06
30 days (n = 4)	0.69 ± 0.21	0.5 ± 0.02
39 days (n = 5)	1.69 ± 0.34	0.8 ± 0.3
		
**10^7 ^CD3^IL-5+^**		
3 days (n = 4)	1.65 ± 0.16	0.6 ± 0.4
7 days (n = 5)	1.12 ± 0.32	0.3 ± 0.1
10 days (n = 4)	2.44 ± 1.0	1.9 ± 1.1
21 days (n = 5)	1.95 ± 0.84	0.8 ± 0.2†
30 days (n = 5)	1.9 ± 0.23†	2.7 ± 0.7†
39 days (n = 4)	19.19 ± 2.0†	21.4 ± 6.8†

BM and blood eosinophil numbers in SCID-bg mice after adoptive transfer of 10^7 ^CD3^IL-5+ ^T lymphocytes in 0.35 ml 0.9% NaCl or 0.9% NaCl alone. Recipients were sacrificed on day 3, 10, 14, 21, 30 or 39 after the cell transfer. Values are shown as mean ± SEM. † p < 0.05 *vs*. respective 0.9% NaCl-injected control group.

### ELISA

Mouse IL-5 levels in serum were detected using commercial murine IL-5 ELISA kit (R&D Systems, Inc, Abingdon, UK). The lower detection limit was 3.9 pg/ml.

### Statistical analysis

All data are expressed as mean ± SEM. Statistical analysis was carried out using a non-parametric analysis of variance (Kruskal-Wallis test) to determine the variance among more than two groups. If significant variance was found, an unpaired two-group test (Mann-Whitney U test) was used to determine significant differences between individual groups. *P *< 0.05 was considered statistically significant.

## Results

### Eosinophils in naïve CD3^IL-5+^, CD3^IL-5+^/CD4^-/- ^and CD3^IL-5+^/CD8^-/- ^mice

#### Bone marrow

**Figure 1:**
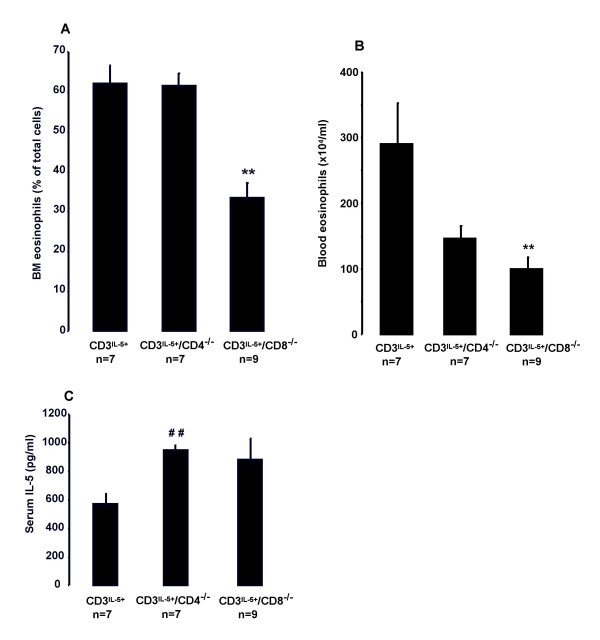
**Eosinophils in naïve CD3^IL-5+^, CD3^IL-5+^/CD4^-/- ^and CD3^IL-5+^/CD8^-/- ^mice**. Eosinophils in A) BM and B) blood of naïve CD3^IL-5+^, CD3^IL-5+^/CD4^-/- ^and CD3^IL-5+^/CD8^-/- ^mice. C) Serum IL-5 in naïve CD3^IL-5+^, CD3^IL-5+^/CD4^-/- ^and CD3^IL-5+^/CD8^-/- ^mice. Data are shown as mean (+SEM) (n = 7–9). **P < 0.01 decreased from CD3^IL-5+ ^mice. ^##^*P *< 0.01 increased from CD3^IL-5+ ^mice.

The number of BM eosinophils was significantly reduced in CD3^IL-5+ ^mice gene knockout for CD8 (CD3^IL-5+^/CD8^-/-^) as compared to CD3^IL-5+^mice and CD3^IL-5+ ^mice gene knockout for CD4 (CD3^IL-5+^/CD4^-/-^) (33 ± 4% *vs*. 62 ± 5% and 62 ± 3% of total cells respectively; P = 0.008, Fig 1A). There was no difference in BM eosinophils when CD3^IL-5+^/CD4^-/- ^and CD3^IL-5+ ^mice were compared (62 ± 5% *vs*. 62 ± 3% of total cells respectively, Fig 1A)

#### Blood

The number of blood eosinophils was significantly reduced in CD3^IL-5+^/CD8^-/- ^as compared to CD3^IL-5+ ^(290 ± 63 *vs*. 100 ± 18 × 10^4^/ml; P = 0.008, Fig. 1B). There was no significant difference in the number of blood eosinophils in the CD3^IL-5+^/CD4^-/- ^when compared to CD3^IL-5+ ^(146 ± 19 *vs*. 290 ± 63 × 10^4^/ml; P = NS, Fig. 1B).

### Serum IL-5 in naïve CD3^IL-5+^, CD3^IL-5+^/CD4^-/- ^and CD3^IL-5+^/CD8^-/- ^mice

There was no significant difference in serum IL-5 between the CD3^IL-5+^/CD8^-/- ^and CD3^IL-5+ ^mice (880 ± 149 *vs*. 573 ± 66 pg/ml, Fig. 1C). Serum IL-5 was significantly increased in CD3^IL-5+^/CD4^-/- ^mice compared to CD3^IL-5+ ^mice (949 ± 34 *vs*. 573 ± 66 pg/ml p = 0.008, Fig. 1C).

### Time-course experiment

A significant increase in blood eosinophils was evident on day 21 after transfer of CD3 cells from naïve CD3^IL-5+ ^to SCID-bg mice. A significant increase in BM eosinophils was not evident until 30 days after the cell transfer. The most pronounced increase in number of blood and BM eosinophils was observed 39 days after the cell transfer (Table 1). There were no time-dependent changes in BM eosinophils in the 0.9% NaCl-injected control groups.

### Eosinophil numbers after adoptive transfer of CD3^IL-5+ ^CD3^+^, CD4^+ ^or CD8^+ ^T cells to SCID-bg mice

#### Bone marrow

**Figure 2:**
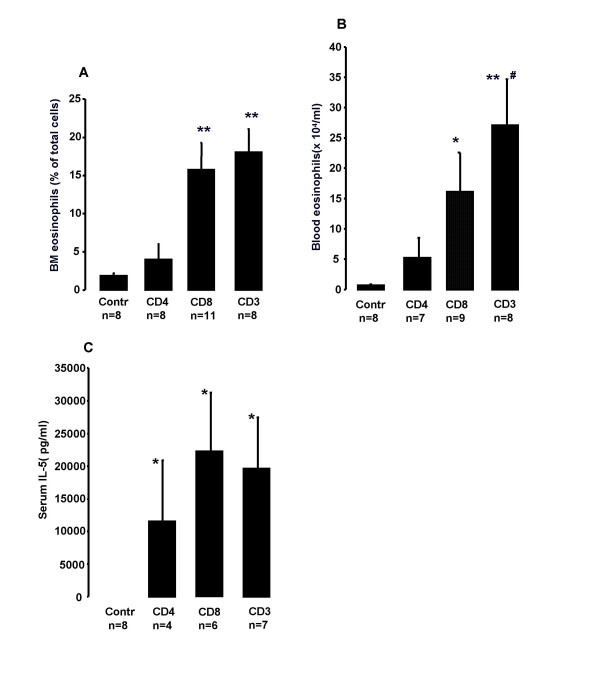
**Eosinophil numbers after adoptive transfer of CD3^IL-5+ ^CD3^+^, CD4^+ ^or CD8^+ ^T cells to SCID-bg mice**. Eosinophils in A) BM and B) blood of naïve SCID-bg mice 39 days after adoptive transfer of CD4^+^, CD8^+ ^and CD3^+ ^T cells enriched from naïve CD3^IL-5+ ^mice. C) Serum IL-5 in SCID-bg mice 39 days after adoptive transfer of CD4^+^, CD8^+ ^and CD3^+ ^T cells enriched from naïve CD3^IL-5+ ^mice. Data are shown as mean (+SEM) (n = 4–11). *P < 0.05 increased from control treated mice. **P < 0.01 increased from control treated mice and mice adoptively transferred with CD4^+ ^cells from naïve CD3^IL-5+ ^mice. ^#^*P *< 0.05 increased from control treated mice and mice adoptively transferred with CD4^+ ^cells from naïve CD3^IL-5+ ^mice.

Transfer of CD3^+ ^T cells from naïve CD3^IL-5+ ^induced an increase in the number of BM eosinophils in SCID-bg mice compared to the 0.9% NaCl-injected control group and transfer of CD3^IL-5+ ^CD4^+ ^T cells (18.01 ± 3.09% *vs*. 1.86 ± 0.35% and 3.96 ± 2.02% of total cells; P = 0.001 and 0.003, respectively Fig. 2A). Transfer of naïve CD3^IL-5+ ^CD8^+ ^T cells induced an increase in the number of BM eosinophils compared to the 0.9% NaCl-injected control group and transfer of CD3^IL-5+ ^CD4^+ ^T cells (15.76 ± 3.51% *vs*. 1.86 ± 0.35% and 3.96 ± 2.02% of total cells; P = 0.002 and 0.006, respectively, Fig. 2A). Transfer of naïve CD3^IL-5+ ^CD4^+ ^T cells did not cause any significant changes in the number of BM eosinophils compared to the 0.9% NaCl-injected control group (1.86 ± 0.35% *vs*. 3.96 ± 2.02% of total cells, Fig. 2A).

#### Blood

Transfer of CD3^IL-5+ ^CD3^+ ^T cells induced blood eosinophilia in SCID-bg mice compared to the 0.9% NaCl-injected control animals and the animals that had been given CD3^IL-5+ ^CD4^+ ^T cells (27 ± 8 *vs*. 0.6 ± 0.2 and 5 ± 3 × 10^4^/ml; P = 0.001 and 0.015, respectively; Fig. 2B). Transfer of CD3^IL-5+ ^CD8^+ ^T cells induced an increase in the number of blood eosinophils in SCID-bg mice compared to the 0.9% NaCl-injected control (16 ± 6 *vs*. 0.6 ± 0.2 × 10^4^/ml; P = 0.038, Fig. 2B). Transfer of CD3^IL-5+ ^CD4^+ ^T cells did not increase blood eosinophilia (5.1 ± 3.3 *vs*. 0.6 ± 0.2 × 10^4^/ml, Fig. 2B)

### Serum IL-5 in SCID-bg mice after adoptive transfer of CD3^IL-5+ ^CD3^+^, CD4^+ ^or CD8^+ ^T cells

Transfer of CD3^IL-5+^CD3^+^, CD4^+ ^and CD8^+ ^splenocytes induced a substantial increase in the concentration of recipient serum IL-5. There were no significant differences in the concentration of serum IL-5 between transfer groups (Fig. 2C).

### Eosinophil numbers after adoptive transfer of C57BL/6 CD3^+^, CD4^+ ^or CD8^+ ^T cells to SCID-bg mice

#### Bone marrow

**Figure 3:**
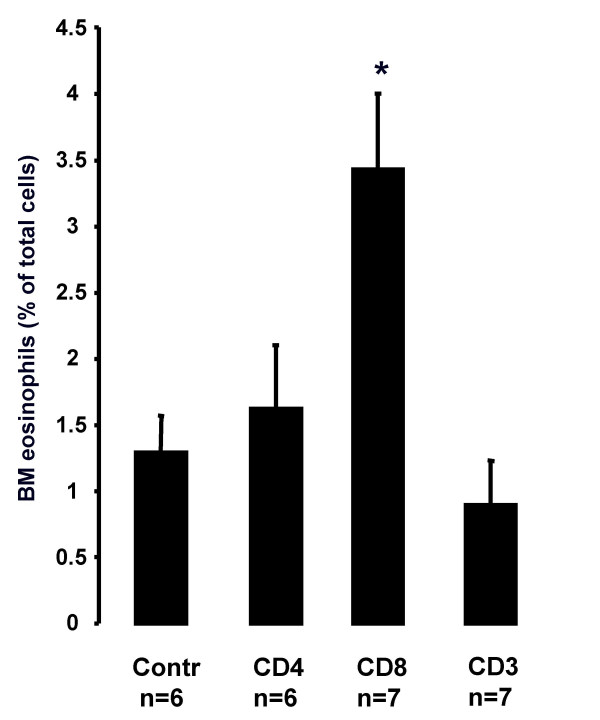
**Eosinophil numbers after adoptive transfer of C57BL/6 CD3^+^, CD4^+ ^or CD8^+ ^T cells to SCID-bg mice**. Eosinophils in BM of naïve SCID-bg mice 39 days after adoptive transfer of CD4^+^, CD8^+ ^and CD3^+ ^T cells enriched from naïve C57BL/6 mice. Data are shown as mean (+SEM) (n = 6–7). *P < 0.05 increased from control treated mice.

Transfer of CD3^+ ^T cells from naïve C57BL/6 mice did not induce BM eosinophilia in SCID-bg mice. Adoptive transfer of CD8^+ ^T cells from naïve C57BL/6 mice induced BM eosinophilia in SCID-bg mice compared to the 0.9% NaCl-injected control group (3.43 ± 0.58% *vs*. 1.29 ± 0.28% of total cells; P = 0.018, Fig. 3). Transfer of CD4^+ ^T cells from naïve C57BL/6 mice did not cause any significant changes in the number of BM eosinophils compared to the 0.9% NaCl-injected control group (1.62 ± 0.48% *vs*. 1.29 ± 0.28% of total cells, Fig. 3).

#### Blood

There was no difference in blood eosinophilia in any of the transferred groups compared to the 0.9% NaCl-injected control mice.

### Newly produced and MBP+ eosinophils in allergen-challenged CD3^IL-5+^, CD3^IL-5+^/CD4^-/- ^and CD3^IL-5+^/CD8^-/- ^mice

#### Bone marrow

**Figure 4:**
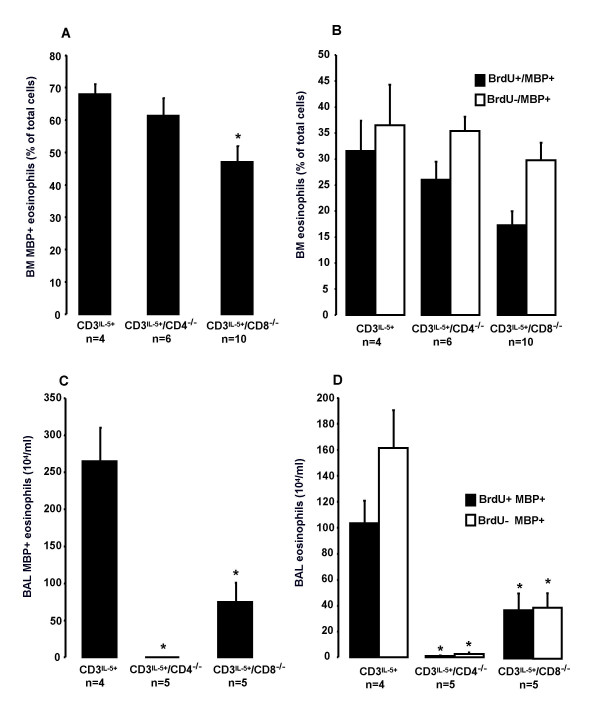
**Newly produced and MBP^+ ^eosinophils in allergen-challenged CD3^IL-5+^, CD3^IL-5+^/CD4^-/- ^and CD3^IL-5+^/CD8^-/- ^mice**. MBP^+ ^eosinophils in A) BM and C) BAL and BrdU^+^/MBP^+ ^eosinophils and BrdU-/MBP^+ ^eosinophils in B) BM and D) BAL of OVA sensitized and exposed CD3^IL-5+^, CD3^IL-5+^/CD4^-/- ^CD3^IL-5+^/CD8^-/- ^mice. Data are shown as mean (+SEM) (n = 4–9). *P<0.05 decreased from CD3^IL-5+ ^mice.

The number of BM MBP^+ ^eosinophils was significantly reduced in the allergen exposed CD3^IL-5+^/CD8^-/- ^mice when compared to the CD3^IL-5+ ^mice (47 ± 3% *vs*. 68 ± 3% of total cells; P = 0.016, Fig 4A). The number of MBP^+ ^eosinophils in CD3^IL-5+^/CD4^-/- ^was not different compared to the CD3^IL-5+ ^mice (61 ± 5% *vs*. 68 ± 3% of total cells; P = NS, Fig 4A). We were not able to detect any significant reduction in the newly produced (BrdU+/MBP+) BM eosinophils in the allergen exposed CD3^IL-5+^/CD8^-/- ^mice when compared to the CD3^IL-5+ ^mice (17 ± 3% *vs*. 32 ± 6% of total cells (P = NS, Fig 4B).

#### BALF

A significant reduction of MBP^+ ^eosinophils was found in both CD3^IL-5+^/CD8^-/- ^and CD3^IL-5+^/CD4^-/- ^mice compared to the CD3^IL-5+ ^mice after allergen challenge (75 ± 26 and 3 ± 2 *vs*. 265 ± 45 × 10^4^/ml BALF; P = 0.028 and P = 0.014 respectively, Fig. 4C). A significant reduction was also found in the newly produced BALF eosinophils (i.e BrdU+/MBP+ cells) in CD3^IL-5+^/CD8^-/- ^and CD3^IL-5+^/CD4^-/- ^mice as compared to CD3^IL-5+ ^mice (37 ± 13 and 1 ± 0.5 *vs*. 104 ± 17 × 10^4^/ml BALF ; P = 0.028 and P = 0.014 respectively, Fig. 4D). However, also the BrdU negative eosinophils (i.e BrdU-/MBP+ cells) were reduced compared to the CD3^IL-5+ ^mice (38 ± 13 and 2 ± 1 *vs*. 161 ± 29 × 10^4^/ml BALF; P = 0.014 and P = 0.014 respectively, Fig. 4D).

## Discussion

This study provides evidence, based on several different experimental approaches, that CD8^+ ^T lymphocytes are intricately involved in the regulation of BM eosinophilopoiesis. Thus, naïve crossbred CD3^IL-5+^/CD8^-/- ^mice showed a significant decrease in the number of BM eosinophils when compared to naïve CD3^IL-5+ ^or naïve crossbred CD3^IL-5+^/CD4^-/- ^mice. Adoptive transfer of CD8^+^, but not CD4^+ ^T lymphocytes from naïve CD3^IL-5+ ^or C57BL/6 wild type mice restored BM eosinophilia in immunodeficient SCID-bg mice. Additionally, allergen exposed CD3^IL-5+^/CD8^-/- ^mice showed a reduced number of BM eosinophils when compared to CD3^IL-5+ ^mice. Both CD3^IL-5+^/CD8^-/- ^and CD3^IL-5+^/CD4^-/- ^mice showed a significant reduction in BALF eosinophils following allergen exposure.

Recent data is suggesting that not only CD4^+ ^T lymphocytes, but also CD8^+ ^T lymphocytes, contribute to allergen-induced airway inflammation. Depletion of CD8^+ ^T lymphocytes prior to allergen challenge has been shown to decrease Th_2 _cytokines, reduce eosinophil recruitment into the airway and reduce airway hyperresponsiveness [19-22]. Although CD4^+ ^and CD8^+ ^T lymphocytes appear to be involved in the regulation of local airway inflammation, less is known about their role in BM eosinophilopoiesis after allergen exposure. The number of CD3^+ ^T lymphocytes expressing IL-5 mRNA and protein is increased in BM, circulation as well as in the airways following allergen challenge in both mice and humans [5,15-17]. Therefore, in the present study we utilized IL-5 transgenic mice (CD3^IL-5+^) that constitutively overexpress IL-5 in CD3^+ ^T lymphocytes [23], which is known to result in an enhanced eosinophilopoiesis and increased levels of circulating eosinophils [7,23]. Importantly, we have recently shown that adoptive transfer of CD3^+ ^T lymphocytes from sensitized CD3^IL-5+ ^mice induced an increase in BM eosinophils in allergen-exposed recipient wild type mice [7].

To assess the role of CD4^+ ^and CD8^+ ^T lymphocytes in BM eosinophilopoiesis we crossbred gene knockout mice deficient in CD4^+ ^or CD8^+ ^T lymphocytes with CD3^IL-5+ ^mice. Notably, CD3^IL-5+ ^mice deficient in CD8^+ ^T lymphocytes had a reduced number of BM eosinophils compared to CD3^IL-5+ ^mice or CD3^IL-5+ ^deficient in CD4^+ ^T lymphocytes. Initially, we hypothesized that this could be due a difference in IL-5 production between the crossbred mice, since CD8^+ ^T lymphocytes can produce several Th_2 _cytokines including IL-5 [19,20]. A significant increase in serum IL-5 levels was found in CD3^IL-5+ ^mice deficient in CD4^+ ^T lymphocytes compared to CD3^IL-5+ ^mice. It could be speculated that this phenomena is due to a lack of T regulatory cells in these mice. However, we were not able to find any difference in serum IL-5 between the two crossbred strains, indicating that CD8^+ ^T lymphocytes are required to maintain high levels of a strongly IL-5 dependent BM eosinophilopoiesis. Importantly, our present study further shows that adoptive transfer of CD3^IL-5+ ^CD8^+ ^T lymphocytes as well as transfer of CD8^+ ^T lymphocytes from C57BL/6 mice restored BM eosinophilia in immunodeficient (SCID-bg) mice. The finding that not only transfer of CD3^IL-5+ ^CD8^+ ^T lymphocytes but also transfer of CD8^+ ^T lymphocytes from C57BL/6 mice restore BM eosinophilia in immunodeficient mice further argues that the role of CD8^+ ^T lymphocytes in BM eosinophilopoiesis is independent of IL-5 overproduction. Importantly, IL-5 is not only produced by CD4^+ ^T lymphocytes, but also CD8^+ ^T lymphocytes, as well as CD34^+ ^cells. The initial development of eosinophilia is induced in a complex way, including T lymphocyte independent mechanisms, as well as production of IL-5 from CD34^+ ^cells [14,24]. CD8^+ ^T lymphocytes probably interact in this process both by IL-5 dependent as well as IL-5 independent mechanisms (Figure 2A and 3, respectively).

In allergen-exposure experiments, we further show that CD8^+ ^T lymphocytes are involved also in allergen-induced BM eosinophilopoiesis. In this experiment, we stained cells with a monoclonal antibody to eosinophil granule major basic protein (MBP), since is known that this is expressed early on eosinophil-committed cells [25,26]. Allergen exposed CD3^IL-5+^/CD8^-/- ^mice showed a reduction of BM MBP^+ ^eosinophils compared to CD3^IL-5+ ^mice, whereas in the CD3^IL-5+^/CD4^-/- ^mice the number of BM MBP^+ ^eosinophils remained unchanged compared to CD3^IL-5+ ^mice. One explanation to this could be a reduced production of eosinophils in the CD3^IL-5+^/CD8^-/- ^mice. We directly addressed this question by using a double staining technique for newly produced eosinophils (i.e. BrdU^+^/MBP^+ ^cells). However, we where not able to show any significant reduction in BrdU^+^/MBP^+ ^BM eosinophils in any of the crossbred strains compared to CD3^IL-5+ ^mice, although the CD3^IL-5+^/CD8^-/- ^mice showed a trend of a reduction in BrdU^+^/MBP^+ ^eosinophils. It could be speculated that the production of eosinophils in the BM has a rapid turnover in these mice and that the newly produced cells are released in to the circulation and already accumulated in the airways.

By contrast, allergen-induced airway BrdU^+^/MBP^+ ^eosinophils were significantly reduced in both CD3^IL-5+^/CD8^-/- ^and CD3^IL-5+^/CD4^-/- ^mice compared to CD3^IL-5+ ^mice. Notably, when CD4^+ ^T lymphocytes were eliminated, almost no recruitment of eosinophils into the airways occurred. However, for the restoration of the allergen-induced eosinophil recruitment into the airways, both CD4^+ ^and CD8^+ ^T lymphocyte subsets may be required, which is in agreement with a recent report [20]. It has been previously shown that CD4^+ ^T lymphocytes are required for traffic of eosinophils to airways, also in mice that excessively overexpress IL-5 in the airway epithelium [27]. Thus, CD4^+ ^T lymphocytes are contributing to eosinophil traffic to airways in parallel to IL-5. However, our present study also shows that when CD8^+ ^T lymphocytes are lacking in a mouse overexpressing IL-5 in CD3^+ ^T lymphocytes, a reduction in the recruitment of eosinophils to the airways occur. This seems to be a reflection of a reduced production of eosinophils in the BM in CD8^+ ^T lymphocyte deficient mice. Furthermore, it has recently been shown that CD8^+ ^T lymphocytes are a source of IL-13 [22]. Therefore depletion of CD8^+ ^T lymphocytes may partly reduce airway eosinophilia as a consequence of a reduction in IL-13, since it has been reported that administration of IL-13, or overexpression of IL-13 in the airways, induces eosinophilia [28,29].

## Conclusion

In summary, we here show for the first time that CD8^+ ^T lymphocytes regulate BM eosinophilopoiesis both at baseline and after allergen exposure. In the presence of IL-5, CD8^+ ^T lymphocytes seem to be required for the maintenance of eosinophil production in the BM, while CD4^+ ^T lymphocytes are required for their recruitment into the airways following airway allergen exposure. Thus, CD8^+ ^T lymphocytes are involved in some of the systemic processes in allergic eosinophilia, which has implications in understanding the overall complex mechanisms of allergic diseases.

## Competing interests

The author(s) declare that they have no competing interests.

## Authors' contributions

MR carried out the cross bred mice experiments and allergen-challenge experiment, design and coordinated the study and wrote the manuscript. SS carried out the SCID-bg mice experiments, design and coordinated the study and participated in writing the manuscript. A-K J carried out the SCID-bg mice experiments, design and coordinated the study and participated in drafting the manuscript. CM carried out the genotyping of cross bred mice. MS participated in the coordination of the study. AB carried out flow cytometry measurements and participated in drafting the manuscript. JJL participated in the coordination of the study. JL conceived the study, and participated in its design and coordination and helped to draft the manuscript.
